# Changes in risk perceptions during the 2014 Ebola virus disease epidemic: results of two consecutive surveys among the general population in Lower Saxony, Germany

**DOI:** 10.1186/s12889-018-5543-1

**Published:** 2018-05-15

**Authors:** Julie Obenauer, Nicole Rübsamen, Ekaterine Garsevanidze, André Karch, Rafael T. Mikolajczyk

**Affiliations:** 1 Department of Epidemiology and Biostatistics, East Tennessee State University, Johnson City, Tennessee USA; 2 Department of Epidemiology, Helmholtz Centre for Infection Research (HZI), Braunschweig, Germany; 3 PhD Programme “Epidemiology”, Braunschweig-Hannover, Germany; 4 German Centre for Infection Research, Hannover-Braunschweig, Germany; 5 Hannover Medical School, Hannover, Germany; 6 Institute for Medical Epidemiology, Biometry, and Informatics (IMEBI), Medical Faculty of the Martin Luther University Halle-Wittenberg, Halle (Saale), Germany

**Keywords:** Ebola virus disease, Knowledge, Risk perception

## Abstract

**Background:**

The Ebola virus disease (EVD) outbreak 2014 received extensive news media coverage, which faded out before the outbreak ended. News media coverage impacts risk perception; it is, however, unclear if the components of risk perception (affective and cognitive responses) change differently over time.

**Methods:**

In an online panel, we asked participants (*n* = 1376) about EVD risk perceptions at the epidemic’s peak (November 2014) and after news media coverage faded out (August 2015). We investigated worry (affective response), perceived likelihood of infection, perceived personal impact, and coping efficacy (dimensions of cognitive response), and knowledge about transmission. Differences between the surveys with respect to manifestations of affective and cognitive dimensions were tested using the Wilcoxon signed-rank test. The association between individual change in knowledge and worries about EVD in the first survey was investigated using linear regression.

**Results:**

In November 2014, the survey was filled in by 974 participants. Ten months later, 662 of them were still members of the online panel and were invited to the follow-up survey. Among the 620 respondents, affective response decreased between the surveys. Knowledge about EVD also decreased; however, participants worried about EVD in 2014 had increased knowledge in 2015. Perceived likelihood of infection decreased over time, while perceived personal impact and coping efficacy did not.

**Conclusions:**

Risk communication appealing to cognitive reactions by informing clearly on the risk of infection in unaffected countries may decrease inappropriate behaviors.

**Electronic supplementary material:**

The online version of this article (10.1186/s12889-018-5543-1) contains supplementary material, which is available to authorized users.

## Background

In December of 2013, the largest Ebola virus disease (EVD) outbreak to date started in Western Africa [1]. This outbreak would cause 28,616 cases with 11,310 deaths before the World Health Organization rescinded the Public Health Emergency designation on March 29, 2016 [2]. During the emergency, news media outlets around the globe covered EVD extensively, often resulting in the spread of more panic than information [3]. However, this reporting reached a peak in late October of 2014 [4], when two developed nations reported local transmission due to infection by cases brought back for treatment [5]. More than a year later, despite continuing transmission in Western Africa, EVD had all but disappeared from the news [4, 6].

News media coverage (in newspapers, radio television, and the Internet) has been shown to impact the risk perceptions of individuals during public health emergencies, i.e. “how they judge and interpret available evidence on their possible losses and the particular vulnerability associated with the risk” [7]. Consumers draw conclusions of severity and likelihood of risk based on the number of reports they see on an issue and the tone and content of those reports [8]. These stories may lead to short-term concern, and even panic, but the effect tapers off over the course of the epidemic [9].

There are, however, two different kinds of risk perceptions (affective and cognitive response [10]) which might show different patterns of change over the course of public health emergencies. While affective response is defined as an emotional response to a risk, cognitive factors include the perceived seriousness of the threat and perceived coping efficacy. Cameron and Leventhal suggested that the affective response is experiential, quick, and intuitive while the cognitive response is deliberate, slow, and rule-based [11].

As the EVD outbreak was such a decisive event for many Germans, we aimed to investigate if inappropriate risk perceptions persisted even after news media coverage had diminished. If so, then public health interventions could help to decrease inappropriate behaviors in case of other emerging infectious disease outbreaks. We specifically investigated if affective and cognitive response showed different patterns of change.

## Methods

### Participants

**Fig. 1:**
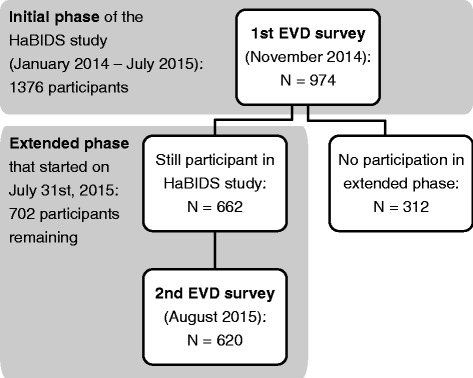
Participant flow diagram

Participants were asked to complete a survey on their risk perceptions regarding EVD at two time points during the epidemic (in November 2014 and in August 2015). We implemented the surveys using a longitudinal online panel that was initiated in March 2014 to address human hygiene and preventive behavior regarding infectious diseases (HaBIDS), which is described in detail elsewhere [12, 13]. In brief, the panel was established using stratified random sampling from the population registry in four districts in Lower Saxony, Germany (Braunschweig, Salzgitter, Vechta, and Wolfenbüttel). Of 26,895 individuals 15–69 years of age (minors under 16 years old were also included) invited to the HaBIDS study, 9% were successfully recruited: 1376 individuals opted for the online panel and 935 individuals opted for paper-based participation. In November 2014, after three patients who had acquired EVD in Western Africa had been evacuated to hospitals in Germany for treatment, all participants in the online panel were invited to fill in a questionnaire about EVD risk perception (paper-based participants were excluded because printing and sending the questionnaires would have taken too long). The response rate to this survey was 71% (*n* = 974 out of 1376, Fig. 1). The initial phase of the HaBIDS panel ended in July 2015. All participants who had not formally withdrawn from the study so far were invited to continue with the study. Half of the online participants (*n* = 702) opted in for this extended phase. In August 2015, all responders to the first EVD survey who were still in the panel (*n* = 662, Fig. 1) were invited to fill in the second survey.

### Measures

The first survey is described in detail elsewhere [14]. Because the questionnaire had to be disseminated quickly to asses risk perceptions immediately after the EVD patients had been evacuated to Germany, no validation or pre-testing of the questionnaire was performed. To investigate change in risk perceptions, several questions were re-asked in the second survey. The questionnaires of both surveys can be found in Additional file 1.

### Affective response

The overall perception of EVD risk was assessed by the yes-or-no question “Are you worried about Ebola?” Participants were also asked if they were worried that in the next 3 months, people might arrive in Germany who are identified as infected persons after their entry, that individual persons might be infected with the Ebola virus in Germany during the next 6 months and that in the next 6 months Ebola could spread in the general population of Germany similar to how it was spreading currently in Western Africa.

### Knowledge

To assess knowledge of EVD (as one factor involved in affective as well as cognitive response [15]), participants were asked “How can Ebola be transmitted?” and given a series of options with the response choices of “yes”, “no”, and “don’t know”. A cumulative knowledge score was computed by assigning one point for each answer in agreement with current scientific knowledge (range 0–11).

### Cognitive response

We assessed three cognitive dimensions of risk perception that have been elaborated by Prati and colleagues [16] in the context of pandemic influenza H1N1 2009: perceived likelihood of infection, perceived personal impact, and coping efficacy.

To assess perceived likelihood of infection, participants were asked to consider the worldwide EVD situation and determine their personal risk of acquiring Ebola in different situations.

Perceived personal impact was measured by asking participants if they would change their behavior if an Ebola patient was evacuated from Africa and brought to Germany for treatment in a near-by hospital.

For coping efficacy, participants responded to items regarding the prevention of spread of EVD in case of individuals coming back from regions with EVD.

### Statistical analysis

To investigate if excluding responders to the first EVD survey who did not participate in the second survey could affect the results, differences in sociodemographic characteristics and risk perception between individuals who responded to both surveys and individuals who responded to the first survey only were tested using Wilcoxon rank-sum test for continuous variables (age, knowledge score) and chi-squared test for categorical variables. The analyses were then restricted to individuals who responded to both surveys. Differences in responses between the first and the second survey were tested using the Wilcoxon signed-rank test. The association between individual differences in knowledge scores (calculated as the difference of knowledge score in 2015 minus knowledge score in 2014) and worries about EVD in the first survey was tested using linear regression, adjusted for age, sex, and education. We conducted complete-case analyses and considered *p* ≤ 0.05 as statistically significant. Analyses were performed using Stata 12 (StataCorp LP, College Station, TX, USA).

## Results

We analyzed data of 620 participants who responded to both surveys. Half of them were female, and the majority had earned a university degree (Table 1). The median age was 49 years.

**Table 1:** Sociodemographic characteristics of the participants in the two consecutive surveys about EVD risk perceptions

	Participants in both surveys (*N* = 620)	Participants in first survey only (*N* = 354)	*p*-value
Age (years), median (interquartile range)	49 (37, 58)	42 (31, 53)	< 0.001
Women, N (%)	362 (58.4%)	189 (54.2%)	0.23
Highest completed educational level, N (% of 604 non-missing answers)			
• Lower secondary education or apprenticeship	168 (27.8%)	75 (21.8%)	0.05
• Still at upper secondary school	8 (1.3%)	7 (2.0%)	0.57
• University entrance qualification (upper secondary education or vocational school)	168 (27.8%)	102 (29.7%)	0.6
• University degree	260 (43.1%)	160 (46.5%)	0.33
Born in Germany, N (% of 602 non-missing answers)	577 (95.8%)	325 (94.5%)	0.42

### Non-responder analysis

Participants who responded to both surveys were slightly older than those who, because of the design of HaBIDS, responded to the first survey only (Table 1); there were no differences with respect to sex, education, and country of birth. Participants who responded to both surveys had a slightly higher knowledge score in the first survey than those who responded to the first survey only (Additional file 2). Dimensions of affective and cognitive response in the first survey did not differ between the two groups of participants (Additional file 2).

### Affective response

The number of participants worried about EVD dropped from 27.3% during the first survey to 2.7% for the second (Table 2). The number of people who were worried that someone would be identified as infected after entering the country dropped by 50 percentage points between the surveys. Additionally, at the height of the outbreak in Western Africa, 3.2% of respondents worried that Germany would experience a similar outbreak. Only 1.3% of individuals reported these worries in the second survey.

**Table 2:** Change in affective response, knowledge, and cognitive response in the two consecutive surveys about EVD

	First survey	Second survey	Difference	*p* value
Affective response				
Percentage of participants who are worried...				
about EVD.	27.3%	2.7%	24.6%	< 0.001
that in the next three months people might arrive in Germany who are identified as infected persons after their entry.	77.0%	27.6%	49.4%	< 0.001
that individual persons might be infected with the Ebola virus in Germany during the next six months.	57.7%	19.7%	38.0%	< 0.001
that in the next six months Ebola could spread in the general population of Germany similar to how it is spreading currently in Western Africa.	3.2%	1.3%	1.9%	0.015
Knowledge				
Percentage of participants who answer correctly				
By direct contact with bodily fluids of infected persons, either dead or living	94.3%	96.1%	−1.8%	0.11
By direct contact with infected, but asymptomatic persons	26.6%	8.4%	18.2%	< 0.001
Through air, if infected people cough or sneeze	27.6%	18.3%	9.3%	< 0.001
Through material which has been heavily contaminated with bodily fluids of dead or living infected persons	84.8%	80.2%	4.6%	0.015
Through drinking water	66.4%	55.1%	11.3%	< 0.001
Through food produced in Germany	96.7%	93.7%	3.0%	0.0068
By casual contact with someone already sick, such as sitting next to the person (without any direct contact of bodily fluids)	59.6%	51.3%	8.3%	< 0.001
By wild animals in Africa (monkeys, bats)	50.3%	55.2%	−4.9%	0.021
By wild animals in Germany (rats, foxes)	79.0%	81.8%	−2.8%	0.14
By insects in Africa (mosquitoes, tsetse flies)	57.9%	53.3%	4.6%	0.053
By insects in Germany (midges)	85.1%	85.4%	−0.3%	0.92
Cognitive response: likelihood of infection				
Percentage of participants who think that they have a personal risk of acquiring Ebola…				
at work.	9.3%	3.9%	5.4%	< 0.001
in public transport.	16.4%	7.4%	9.0%	< 0.001
in public places (school, childcare …) or public events.	16.6%	8.5%	8.1%	< 0.001
at an airport in Germany.	37.4%	21.6%	15.8%	< 0.001
as a patient in a German hospital.	14.8%	8.9%	5.9%	< 0.001
at a doctor’s office in Germany.	16.2%	8.1%	8.1%	< 0.001
during a travel to affected countries.	72.3%	57.3%	15.0%	< 0.001
Cognitive response: personal impact				
Percentage of participants who would…				
avoid public events and crowded places.	15.1%	14.6%	0.5%	0.77
avoid using public transport.	14.7%	13.1%	1.6%	0.29
avoid physical contact with other people.	33.9%	30.3%	3.6%	0.086
pay more attention to hygiene (e.g. wash hands more often).	66.6%	61.0%	5.6%	0.0091
wear a face mask outside of my home.	1.1%	1.8%	−0.7%	0.33
not want to be admitted to the same hospital.	49.4%	42.5%	6.9%	< 0.001
not visit friends admitted to the same hospital.	26.8%	20.9%	5.9%	0.0024
Cognitive response: coping efficacy				
Percentage of participants who support specific measure to prevent the spread of EVD to Europe				
Provide information on EVD for all travellers coming from affected areas and advice in case of developing symptoms	97.2%	93.6%	3.6%	0.0018
Get personal information of all travellers coming from affected areas and control their health three weeks long	68.9%	49.9%	19.0%	< 0.001
Forbid return transport of Germans getting infected during assistance intervention in Western Africa	8.5%	8.6%	−0.1%	0.9
Forbid bringing EVD patients for treatment to Germany	24.8%	23.3%	1.5%	0.43
Measure temperature of all travellers coming from affected countries when they arrive in Europe with subsequent quarantine for those with high temperature	58.4%	39.2%	19.2%	< 0.001
Measure temperature of all travellers coming from affected countries when they leave Africa with subsequent quarantine for those with high temperature	53.3%	41.4%	11.9%	< 0.001
Mandatory quarantine for all volunteers returning from assistance intervention in Western Africa	38.5%	28.4%	10.1%	< 0.001
Visa ban for people from affected countries	16.9%	7.7%	9.2%	< 0.001
Forbid traveling from Germany to affected countries in Africa	15.5%	8.3%	7.2%	< 0.001
Compulsory vaccination against Ebola for all inhabitants of affected countries as soon as a vaccine is available	86.6%	88.3%	−1.7%	0.29

### Knowledge

Overall knowledge of routes of EVD transmission decreased slightly between the first survey and the follow-up survey. Most noticeable was the decrease in respondents answering correctly that EVD could not be spread by direct contact with asymptomatic individuals (Table 2). During the first survey, 26.6% of respondents answered this question correctly, but that decreased to 8.4% at follow-up (*p* < 0.001). Significantly more respondents answered the question about EVD being spread by infected animals in Africa correctly in the follow-up survey (55.2%) than in the first survey (50.3%).

In the adjusted linear regression analysis, the individual difference in knowledge score was associated with being worried about EVD in 2014 (beta = 0.48, 95% confidence interval [0.09, 0.86], *p* = 0.015) with no influence of age, sex, or education (Table 3).

**Table 3:** Linear regression of knowledge change in the two consecutive surveys about EVD risk perceptions

	Beta (95% CI)	p-value
Worried about EVD during first survey		0.015
• No	Reference	
• Yes	0.48 (0.09, 0.86)	
Sex		0.22
• Male	Reference	
• Female	0.22 (−0.13, 0.57)	
Age (per 1 year increase)	−0.01 (− 0.02, 0.001)	0.082
Education		0.21
• Lower secondary education or apprenticeship	0.40 (−1.14, 1.95)	
• Still at upper secondary school	0.42 (−0.002, 0.84)	
• University entrance qualification	0.04 (−0.38, 0.46)	
• University degree	Reference	

### Cognitive response

Perceived likelihood of infection decreased in every proposed situation (Table 2). The largest decrease was in feeling at risk of contracting EVD at an airport in Germany, which decreased by 15.8 percentage points, followed by contracting the virus during travel to affected countries, which decreased by 15.0 percentage points.

Changes in perceived personal impact were less marked and many participants reported similar behavior about if a patient were admitted to a nearby hospital in both surveys (Table 2). The largest change was in the percentage of participants who would not want to be admitted to the same hospital. During the first survey, 49.4% reported that they would not want to be admitted to the same hospital while 42.5% participants responded the same way for the second survey (*p* < 0.001).

During both surveys, the percentages who felt that individuals entering the country should be given information on EVD was above 90% (Table 2). This dropped from 97.2% during the first survey to 93.6% for the follow up, which was statistically significant (*p* = 0.0018). Slightly more respondents felt at follow up that vaccination for inhabitants of affected countries should be mandatory if a vaccine was made available, as compared to the first survey, but the difference was not significant. There was no significant change in the feeling that Germans who were infected during aid missions and patients with EVD should be banned from entering the country; the support of mandatory quarantine and visa bans for persons from affected countries as well as of forbidding travel to affected countries decreased significantly.

## Discussion

Our research showed that affective response as well as knowledge and the cognitive dimension “perceived likelihood of infection” decreased as the epidemic became less visible, while the cognitive dimensions “perceived personal impact” and “coping efficacy” did not.

During the first survey, more than one quarter of participants were worried personally about EVD, but at the second survey, only 2% were, which is directly proportional to the drop in the number of EVD cases; however, over half of the participants felt they were at high risk of contracting EVD if they traveled to an affected country, which implies that participants thought that they would not be able to cope with the situation in affected countries, although they had good knowledge about transmission of EVD and, thus, also how to avoid it. This could cause inappropriate behaviors in case of a new emerging infectious disease outbreak, so that public health campaigns should strengthen people’s coping efficacy.

Overall knowledge about EVD decreased slightly between survey one and survey two. There were fewer correct answers for all questions during survey two; this implies that it was likely that participants had absorbed incorrect information over time. For example, far more people answered that EVD can be spread by asymptomatic persons in the second survey than the first survey.

Looking at knowledge change as a function of worry about EVD we found that individuals who were worried about EVD at survey one were actually likely to answer more transmission questions correctly at survey two than they did during survey one. This may indicate that concern about EVD prompted knowledge seeking.

The change in knowledge may explain why there were no significant changes in responses to questions regarding the import of EVD patients into Germany for treatment. The perceived personal impact might not be influenced by the actual probability of a scenario, but rather by the perceived personal probability of getting infected that people attribute to this scenario.

Support for mandatory vaccination in affected countries as soon as a vaccine becomes available actually increased, with almost 90% of Germans supporting the idea during survey two. It is unknown if this increase reflects the improvements made in vaccine development within this year, but it may be problematic since the EVD outbreak occurred in low-income countries and weakened the health infrastructure that would be needed to distribute the vaccination. Support for the advanced control measures, such as visa bans from affected countries and forbidding Germans from traveling to those countries, did decrease. This change may reflect the decrease in individuals who felt that Germany was at risk of its own outbreak or that they were likely to contract EVD while engaging in daily activities within the national borders.

Overall, people were less willing to call on advanced control measures in the second survey. This likely reflects the diminishing perception of risk as the epidemic received less news media coverage and as the few patients that were in Germany either died or recovered.

Typically, risk perceptions are evaluated during the actual outbreak or in terms of general attitudes to a present risk. In contrast, we focused on the individual persistence of risk perceptions. The strength of our study is that we could survey the same individuals at both time points, which allowed for analyses of individual differences. Other studies, e.g. during the 2009 influenza A(H1N1) outbreak in Hong Kong [17], relied on consecutive cross-sectional surveys with different participants, thus not allowing the analysis of individual predictors. In the context of the EVD outbreak, Jalloh and colleagues investigated knowledge, attitudes, and practices related to EVD in Guinea 3 months before [18] and at the end of the outbreak [19], but they had also to rely on cross-sectional surveys. To our knowledge, the present study is the first to show changes in risk perception in an unaffected country among the same individuals from the peak of the outbreak to its end.

Interestingly, we could show that some perceptions are stable, while others depend apparently on the intensity or actuality of the events. Unfortunately, the more stable perceptions are at the same time those governing behaviors, thus the gap between actual knowledge and (expected) behavior increases. Similar mechanisms can be source of prejudices existing in a population and demonstrate the difficulty of changing behaviors through education or providing information.

One limitation of our study is that risk perception was not measured before November 2014 so we could not assess if the rule-based dimensions of cognitive response were already at a similar level before the EVD outbreak or if they were formed de novo. Due to the design of the HaBIDS study, the sample size decreased from the first to the second survey. However, as we show in Table 1 and Additional file 2, this decrease in sample size had only minimal impact on the composition of the sample. Finally, individuals holding a university degree were overrepresented among the respondents compared to the general population of Lower Saxony, and possibly the respondents were those more interested in the studied questions, which limits the generalizability.

## Conclusions

The quick and intuitive affective response, and also the more intuitive perceived likelihood of infection changed over the course of the epidemic while the slow and rule-based dimensions of cognitive response remained stable, even though the threat had disappeared. These findings underline the importance of providing clear and easily accessible information about the actual risk of acquiring pandemic diseases in case of infections imported to a non-affected country. Once individuals integrate these information into the rules that they base their cognitive response on, the probability of inappropriate or unjustified behavioral changes during epidemics might decrease.

## Additional files

Additional file 1: Questionnaires used in the two consecutive surveys about EVD (PDF 239 kb)

Additional file 2: Differences in 2014’s risk perception between individuals who responded to both surveys and individuals who responded to the first survey only (PDF 209 kb)

## Abbreviations

EVD: Ebola virus disease
HaBIDS: Hygiene and Behaviour Infectious Diseases Study
