# Supplementary material for: Changes in risk perceptions during the 2014 Ebola virus disease epidemic: results of two consecutive surveys among the general population in Lower Saxony, Germany
Source: BMC Public Health. 2018 May 15;18:628. doi: 10.1186/s12889-018-5543-1 (PMC5952518; doi:10.1186/s12889-018-5543-1)
Supplement: Supplementary file 1 — Questionnaires used in the two consecutive surveys about EVD (PDF 239 kb) [file 12889_2018_5543_MOESM1_ESM.pdf]

Article title:

Changes in risk perceptions during the 2014 Ebola virus disease epidemic:

results of two consecutive surveys among the general population in Lower Saxony, Germany

Authors: Julie Obenauer; Nicole Rübsamen; Ekaterine Garsevanidze; André Karch; Rafael T. Mikolajczyk

Journal: BMC Public Health

Additional file 1 – English translation of the questionnaires
(German questionnaires translated into English; English versions not validated for use in surveys)

Each page shows the questions of the 2014 survey on the left and the respective questions of the 2015 survey on the right.

▼ indicates that the question is displayed only if the respective condition is met.
[] Variable names are written in square brackets. They are not visible for the participants.

2014: Ebola risk perception

In March 2014, the first cases of Ebola virus disease were notified in Guinea (West Africa). In the following months, the disease spread to Sierra Leone, Liberia, Nigeria, and Senegal. The WHO declared this outbreak as a Public Health Emergency of International Concern.

We are interested in your thoughts about this outbreak and if/how Ebola affects your daily life in Germany.

Ebola virus disease

[angst1] Are you worried about Ebola?
☐ Yes
☐ No

[angst2] How much are you worried about Ebola?
Please mark a number from 1=”not much” to 5=”very much”
▼ angst1 == “Yes”

Not much				Very much
1	2	3	4	5
☐	☐	☐	☐	☐

2015: Ebola risk perception

In November 2014, we asked you what you think about the Ebola outbreak in West Africa and whether / how Ebola affects your everyday life. We would now like to examine whether these aspects have changed over time. That is why we ask you a few questions from November, some of them in changed form.

Ebola virus disease

[angst1_2015] Are you currently worried about Ebola?
☐ Yes
☐ No

[angst_reason_2015] Why are you worried?
(multiple choice possible)
▼ angst1_2015 == “Yes”
☐ Worried to get infected with Ebola yourself
☐ Worried that a family members gets infected with Ebola
☐ Worried that the outbreak could turn into a pandemic
☐ Worried that many people die in the world
☐ Other reason, namely:

[angst2_2015] How much are you worried about Ebola?
Please mark a number from 1=”not much” to 5=”very much”
▼ angst1_2015 == “Yes”

Not much				Very much
1	2	3	4	5
☐	☐	☐	☐	☐

[angst1_2014] At the time of the last survey (November 2014): Were you worried about Ebola?
☐ Yes
☐ No

[angst_reason_2014] Why were you worried at that time?
(multiple choice possible)
▼ angst1_2014 == “Yes”
☐ Worried to get infected with Ebola yourself
☐ Worried that a family members gets infected with Ebola
☐ Worried that the outbreak could turn into a pandemic
☐ Worried that many people die in the world
☐ Other reason, namely:

2014: Ebola virus disease (continued)

[wissen1] How do you rate your personal knowledge about Ebola virus disease?

- ☐ Very poor
- ☐ Poor
- ☐ Moderate
- ☐ Good
- ☐ Very good

[wissen2] How do you rate the information that you get from the media about the situation in African countries affected by Ebola?

- ☐ Very poor
- ☐ Poor
- ☐ Moderate
- ☐ Good
- ☐ Very good

2015: Ebola virus disease (continued)

[wissen1_2015] How do you rate your current personal knowledge about Ebola virus disease?

- ☐ Very poor
- ☐ Poor
- ☐ Moderate
- ☐ Good
- ☐ Very good

[wissen2_2015] How do you rate the current information that you get from the media about the situation in African countries affected by Ebola?

- ☐ Very poor
- ☐ Poor
- ☐ Moderate
- ☐ Good
- ☐ Very good

2014: Probability of acquiring Ebola

[knowledge] How can Ebola be transmitted?

	Yes	No	Don't know
By direct contact with bodily fluids of infected persons, either dead or living	☐	☐	☐
By direct contact with infected, but asymptomatic persons	☐	☐	☐
Through air, if infected people cough or sneeze	☐	☐	☐
Through material which has been heavily contaminated with bodily fluids of dead or living infected persons	☐	☐	☐
Through drinking water	☐	☐	☐
Through food produced in Germany	☐	☐	☐
By casual contact with someone already sick, such as sitting next to the person (without any direct contact of bodily fluids)	☐	☐	☐
By wild animals in Africa (monkeys, bats)	☐	☐	☐
By wild animals in Germany (rats, foxes)	☐	☐	☐
By insects in Africa (mosquitoes, tsetse flies)	☐	☐	☐
By insects in Germany (midges)	☐	☐	☐

2015: Probability of acquiring Ebola

[knowledge_2015] How can Ebola be transmitted?

	Yes	No	Don't know
By direct contact with bodily fluids of infected persons, either dead or living	☐	☐	☐
By direct contact with infected, but asymptomatic persons	☐	☐	☐
Through air, if infected people cough or sneeze	☐	☐	☐
Through material which has been heavily contaminated with bodily fluids of dead or living infected persons	☐	☐	☐
Through drinking water	☐	☐	☐
Through food produced in Germany	☐	☐	☐
By casual contact with someone already sick, such as sitting next to the person (without any direct contact of bodily fluids)	☐	☐	☐
By wild animals in Africa (monkeys, bats)	☐	☐	☐
By wild animals in Germany (rats, foxes)	☐	☐	☐
By insects in Africa (mosquitoes, tsetse flies)	☐	☐	☐
By insects in Germany (midges)	☐	☐	☐

2014: Probability of acquiring Ebola (continued)

[risk] If you think of the recent worldwide situation about Ebola: Do you think that you have a personal risk of acquiring Ebola…

	Highly likely	Quite likely	Quite unlikely	Highly unlikely	Does not apply
… at work?	☐	☐	☐	☐	☐
... in public transport?	☐	☐	☐	☐	☐
... in public places (school, childcare …) or public events?	☐	☐	☐	☐	☐
… at an airport in Germany?	☐	☐	☐	☐	☐
… as a patient in a German hospital?	☐	☐	☐	☐	☐
… at a doctor's office in Germany?	☐	☐	☐	☐	☐
… during a travel to affected countries?	☐	☐	☐	☐	☐
… by food imported from Western African countries?	☐	☐	☐	☐	☐
... by other products originating in West Africa?	☐	☐	☐	☐	☐

2015: Probability of acquiring Ebola (continued)

[risk_2015] If you think of the recent worldwide situation about Ebola: Do you think that you have a personal risk of acquiring Ebola…

	Highly likely	Quite likely	Quite unlikely	Highly unlikely	Does not apply
… at work?	☐	☐	☐	☐	☐
... in public transport?	☐	☐	☐	☐	☐
... in public places (school, childcare …) or public events?	☐	☐	☐	☐	☐
… at an airport in Germany?	☐	☐	☐	☐	☐
… as a patient in a German hospital?	☐	☐	☐	☐	☐
… at a doctor's office in Germany?	☐	☐	☐	☐	☐
… during a travel to affected countries?	☐	☐	☐	☐	☐
… by food imported from Western African countries?	☐	☐	☐	☐	☐
... by other products originating in West Africa?	☐	☐	☐	☐	☐

2014: Probability of acquiring Ebola (continued)

[pandemie] Are you worried that…

	Highly likely	Quite likely	Quite unlikely	Highly unlikely
… in the next three months people might arrive in Germany who are identified as infected persons after their entry?	☐	☐	☐	☐
… individual persons might be infected with the Ebola virus in Germany during the next six months?	☐	☐	☐	☐
… in the next six months Ebola could spread in the general population of Germany similar to how it is spreading currently in West Africa?	☐	☐	☐	☐

2015: Probability of acquiring Ebola (continued)

[pandemie_2015] Are you worried that…

	Highly likely	Quite likely	Quite unlikely	Highly unlikely
… in the next three months people might arrive in Germany who are identified as infected persons after their entry?	☐	☐	☐	☐
… individual persons might be infected with the Ebola virus in Germany during the next six months?	☐	☐	☐	☐
… in the next six months Ebola could spread in the general population of Germany similar to how it is spreading currently in West Africa?	☐	☐	☐	☐

2014: Personal behavior and prevention measures

[travel1] Imagine that you have booked a flight for the coming week to travel to any of the affected countries in West Africa. What would you do?

- ☐ Take the flight.
- ☐ Cancel the flight, but only if I am paid back my money.
- ☐ Cancel the flight even if 100% of the travel costs would be lost.
- ☐ Cancel the flight, but only to a maximum loss of …% of the travel costs.

[travel2] Imagine that you have booked a flight for the coming week to a non-affected country in Africa. What would you do?

- ☐ Take the flight.
- ☐ Cancel the flight, but only if I am paid back my money.
- ☐ Cancel the flight even if 100% of the travel costs would be lost.
- ☐ Cancel the flight, but only to a maximum loss of …% of the travel costs.

2015: Personal behavior and prevention measures

[travel1_2015] Consider the following scenario: You have won a trip to Africa. You like Africa as a destination and you are happy about winning.
If you are thinking of the current global situation regarding Ebola: Would you take this trip if the trip would go to one of the affected areas in West Africa?

- ☐ Yes
- ☐ No
- ☐ Don’t know

[travel2_2015] Consider the following scenario: You have won a trip to Africa. You like Africa as a destination and you are happy about winning.
If you are thinking of the current global situation regarding Ebola: Would you take this trip if the trip would go to non-affected parts of Africa?

- ☐ Yes
- ☐ No
- ☐ Don’t know

[travel_result] Between March 2014 and today: Did you actually cancel a journey to Africa because of Ebola?

- ☐ Yes, I cancelled a journey to Africa due to the Ebola outbreak.
- ☐ In the period from March 2014 until today, I did not plan / undertake any trip to Africa at all.
- ☐ I have made the trip to Africa as planned.

[travel_destination] In which African country {if(travel_result == “made the trip”, “did you travel”, “did you plan to travel”)}?
▼ travel_result == “cancelled” | travel_result == “made the trip”
enter your answer here

2014: Personal behavior and prevention measures (continued)

[action_change] Did you change your behavior because of the Ebola outbreak in West Africa?

	Yes	No	Does not apply
I engaged in precautionary purchases.	☐	☐	☐
I avoid contact to African people from acquaintanceship.	☐	☐	☐
I avoid contact to African people in public places.	☐	☐	☐
I avoid going to public events (football, theater …).	☐	☐	☐
I engaged in precautionary purchases.	☐	☐	☐

2014: Personal behavior and prevention measures (continued)

[action_hospital] Would you change your behavior if an Ebola patient was evacuated from Africa and brought to Germany for treatment in a near-by hospital?

	Yes	Rather yes	Rather no	No
I would avoid public events and crowded places.	☐	☐	☐	☐
I would avoid using public transport.	☐	☐	☐	☐
I would avoid physical contact with other people.	☐	☐	☐	☐
I would increase my hygiene behavior (e.g. wash my hands more often)	☐	☐	☐	☐
I would wear a face mask outside of my home.	☐	☐	☐	☐
I would not want to be admitted to the same hospital.	☐	☐	☐	☐
I would not visit friends admitted to the same hospital.	☐	☐	☐	☐

2015: Personal behavior and prevention measures (continued)

[action_hospital_2015] Would you change your behavior if an Ebola patient was evacuated from Africa and brought to Germany for treatment in a near-by hospital?

	Yes	Rather yes	Rather no	No
I would avoid public events and crowded places.	☐	☐	☐	☐
I would avoid using public transport.	☐	☐	☐	☐
I would avoid physical contact with other people.	☐	☐	☐	☐
I would increase my hygiene behavior (e.g. wash my hands more often)	☐	☐	☐	☐
I would wear a face mask outside of my home.	☐	☐	☐	☐
I would not want to be admitted to the same hospital.	☐	☐	☐	☐
I would not visit friends admitted to the same hospital.	☐	☐	☐	☐

2014: Personal behavior and prevention measures (continued)

[measure] Should the following measures be introduced to prevent the spread of Ebola to Europe?

	Not on any account	Not encouraged	Encouraged	Yes, absolutely
Provide information on Ebola to all travelers coming from affected areas and provide advice in case one develops signs and symptoms	☐	☐	☐	☐
Get personal information of all travelers coming from affected areas and control their health for three weeks long upon arrival	☐	☐	☐	☐
Forbid return transport for Germans who get infected during aid missions in West Africa	☐	☐	☐	☐
Forbid bringing Ebola patients for treatment to Germany	☐	☐	☐	☐
Measure temperature for all travelers coming from affected countries upon arrival at Europe with subsequent quarantine for those with high temperature	☐	☐	☐	☐
Measure temperature for all travelers coming from affected countries when they are about to leave Africa with subsequent quarantine for those with high temperature	☐	☐	☐	☐
Three weeks of mandatory quarantine for all volunteers returning from aid missions in West Africa	☐	☐	☐	☐
Entry restrictions for people from affected countries	☐	☐	☐	☐
Forbid travelling from Germany to affected countries in Africa	☐	☐	☐	☐
Compulsory vaccination against Ebola for all inhabitants of affected countries as soon as a vaccine is available	☐	☐	☐	☐

2015: Personal behavior and prevention measures (continued)

[measure_2015] Should the following measures be introduced to prevent the spread of Ebola to Europe?

	Not on any account	Not encouraged	Encouraged	Yes, absolutely
Provide information on Ebola to all travelers coming from affected areas and provide advice in case one develops signs and symptoms	☐	☐	☐	☐
Get personal information of all travelers coming from affected areas and control their health for three weeks long upon arrival	☐	☐	☐	☐
Forbid return transport for Germans who get infected during aid missions in West Africa	☐	☐	☐	☐
Forbid bringing Ebola patients for treatment to Germany	☐	☐	☐	☐
Measure temperature for all travelers coming from affected countries upon arrival at Europe with subsequent quarantine for those with high temperature	☐	☐	☐	☐
Measure temperature for all travelers coming from affected countries when they are about to leave Africa with subsequent quarantine for those with high temperature	☐	☐	☐	☐
Three weeks of mandatory quarantine for all volunteers returning from aid missions in West Africa	☐	☐	☐	☐
Entry restrictions for people from affected countries	☐	☐	☐	☐
Forbid travelling from Germany to affected countries in Africa	☐	☐	☐	☐
Compulsory vaccination against Ebola for all inhabitants of affected countries as soon as a vaccine is available	☐	☐	☐	☐

2014: Personal commitment

[help1] Would you volunteer to fight Ebola in West Africa if your experience and knowledge were needed and if your personal situation and your health allowed so?

☐ Yes ☐ Unlikely
☐ Likely ☐ No
☐ Don’t know

[help2] What would be the main reason not to volunteer?

☐ I would be worried about getting infected.
☐ I think that the help would not be useful.
☐ I would be afraid to be overwhelmed by the situation on site.
☐ I would be worried that I might not return to Germany if I get infected.
☐ I would be worried about not being able to get back to Germany because of an entry restriction.
☐ I think every country should solve its problems by itself without depending on help from other countries.
☐ Other reason, namely:

2015: Personal commitment

[help1_2015] Suppose there was another major outbreak of an infectious disease in Africa: Would you go there to help?

☐ Yes ☐ Unlikely
☐ Likely ☐ No
☐ Don’t know

[help1_1_2015] Do you think that your experience/knowledge would be helpful to do that?

☐ Yes ☐ Unlikely
☐ Likely ☐ No
☐ Don’t know

[help1_2_2015] Would your personal situation allow you to go and help in Africa?
(multiple choice possible)

☐ Yes
☐ No, I cannot go because of my family
☐ No, I cannot go because of my job
☐ Don't know
☐ Other (please specify):

[help2_2015] What would be the main reason not to volunteer?

☐ I would be worried about getting infected.
☐ I think that the help would not be useful.
☐ I would be afraid to be overwhelmed by the situation on site.
☐ I would be worried that I might not return to Germany if I get infected.
☐ I would be worried about not being able to get back to Germany because of an entry restriction.
☐ I think every country should solve its problems by itself without depending on help from other countries.
☐ Other reason, namely:

[help3_2015] Do you know someone who has helped in Africa during the Ebola outbreak?

☐ Yes
☐ No

2014: Personal commitment (continued)

[money1] Would you be willing to donate for the fight against Ebola in Africa?

- ☐ Yes
- ☐ Unlikely
- ☐ Likely
- ☐ No
- ☐ Don't know

[money2] Which sum would you donate?
If you have already donated: How much have you donated?
▼ money1 != "No"

- ☐ Up to 10 Euros
- ☐ 11 to 20 Euros
- ☐ 21 to 50 Euros
- ☐ 51 to 100 Euros
- ☐ 101 to 200 Euros
- ☐ More than 200 Euros

[money3] Would you support a nonrecurring, compulsory, and income-related payment for the fight against Ebola in Africa?

- ☐ Yes
- ☐ Unlikely
- ☐ Likely
- ☐ No
- ☐ Don't know

[money4] Which sum would you pay for the cause?
Please enter as a percentage of your monthly net income.
▼ money3 != "No"
enter your answer here

2015: Personal commitment (continued)

[money1_2015] Would you be willing to donate for the fight against Ebola in Africa?

- ☐ Yes
- ☐ Unlikely
- ☐ Likely
- ☐ No
- ☐ Don't know

[money2_2015] Which sum would you donate?
If you have already donated: How much have you donated?
▼ money1_2015 != "No"

- ☐ Up to 10 Euros
- ☐ 11 to 20 Euros
- ☐ 21 to 50 Euros
- ☐ 51 to 100 Euros
- ☐ 101 to 200 Euros
- ☐ More than 200 Euros

[money3_2015] Would you support a nonrecurring, compulsory, and income-related payment for the fight against Ebola in Africa?

- ☐ Yes
- ☐ Unlikely
- ☐ Likely
- ☐ No
- ☐ Don't know

[money4_2015] Which sum would you pay for the cause?
Please enter as a percentage of your monthly net income.
▼ money3_2015 != "No"
enter your answer here

2014: Vaccination

[vaccination1] If a vaccine against Ebola existed, would you opt for the vaccination even if you do not plan to visit affected countries in West Africa and do not have contact with Ebola patients ever?
☐ Yes
☐ No
☐ Don't know

[vaccination2] Would you still do so if the vaccine was associated with occasional mild side effects?
▼ vaccination1 != "No"
☐ Yes
☐ No
☐ Don't know

[vaccination3] Would you still do so if the vaccine was associated with rare and severe side effects?
▼ vaccination1 != "No"
☐ Yes
☐ No
☐ Don't know

[vaccination4] Should there be a compulsory vaccination against Ebola for the medical staff in Germany?
☐ Yes
☐ No
☐ Don't know

[vaccination5] Should there be a compulsory vaccination against Ebola for the general population in Germany if the number of Ebola cases in Germany increased?
☐ Yes
☐ No
☐ Don't know

[vaccination6] A vaccine must be tested for safety and efficacy. In which country should this be done?

	Yes	No	Don't know
In the country that develops the vaccine	☐	☐	☐
In the affected countries of West Africa	☐	☐	☐

2015: Vaccination

[vaccination1_2015] If a vaccine against Ebola existed, would you opt for the vaccination even if you do not plan to visit affected countries in West Africa and do not have contact with Ebola patients ever?
☐ Yes
☐ No
☐ Don't know

[vaccination2_2015] Would you still do so if the vaccine was associated with occasional mild side effects?
▼ vaccination1_2015 != "No"
☐ Yes
☐ No
☐ Don't know

[vaccination3_2015] Would you still do so if the vaccine was associated with rare and severe side effects?
▼ vaccination1_2015 != "No"
☐ Yes
☐ No
☐ Don't know

[vaccination4_2015] Should there be a compulsory vaccination against Ebola for the medical staff in Germany?
☐ Yes
☐ No
☐ Don't know

[vaccination5_2015] Should there be a compulsory vaccination against Ebola for the general population in Germany if the number of Ebola cases in Germany increased?
☐ Yes
☐ No
☐ Don't know

2014: Activities about Ebola

[info] Have you changed your media use since the Ebola outbreak has become public?

	Yes	No
I use the Internet more often/in addition to inform myself about Ebola.	☐	☐
I use television more often/in addition to inform myself about Ebola.	☐	☐
I use the radio more often/in addition to inform myself about Ebola.	☐	☐
I use print media more often/in addition to inform myself about Ebola.	☐	☐
I use information from public institutions (e.g. Robert Koch-Institute, World Health Organization) more often/in addition to inform myself about Ebola.	☐	☐

[activity] Did you do something among the following list of actions in connection to Ebola?
(multiple choice possible)

☐ Write a letter to the editor or a blog entry about Ebola in a paper or on the Internet
☐ Write a comment to an article in the Internet (e.g. Tagesschau.de, Spiegel online)
☐ Discussion in the circle of acquaintances
☐ Adhered to preventive measures at work
☐ Donations
☐ Participation in information events
☐ Organizing an information event
☐ Offer to participate in aid missions in Africa
☐ Offer to help in Germany

[contact] At your workplace, do you have contact to…?

	Yes	No
… patients or persons in need of care?	☐	☐
… more than 10 persons a day (e.g. students, customers, colleagues)?	☐	☐

2015: Activities about Ebola

[activity_2015] Did you do something among the following list of actions in connection to Ebola?
(multiple choice possible)

☐ Write a letter to the editor or a blog entry about Ebola in a paper or on the Internet
☐ Write a comment to an article in the Internet (e.g. Tagesschau.de, Spiegel online)
☐ Discussion in the circle of acquaintances
☐ Adhered to preventive measures at work
☐ Donations
☐ Participation in information events
☐ Organizing an information event
☐ Offer to participate in aid missions in Africa
☐ Offer to help in Germany
