# Supplementary material for: Changes in risk perceptions during the 2014 Ebola virus disease epidemic: results of two consecutive surveys among the general population in Lower Saxony, Germany
Source: BMC Public Health. 2018 May 15;18:628. doi: 10.1186/s12889-018-5543-1 (PMC5952518; doi:10.1186/s12889-018-5543-1)
Supplement: Supplementary file 2 — Differences in 2014’s risk perception between individuals who responded to both surveys and individuals who responded to the first survey only (PDF 209 kb) [file 12889_2018_5543_MOESM2_ESM.pdf]

Article title:
Changes in risk perceptions during the 2014 Ebola virus disease epidemic: results of two consecutive surveys among the general population in Lower Saxony, Germany

Authors:
Julie Obenauer; Nicole Rübsamen; Ekaterine Garsevanidze; André Karch; Rafael T. Mikolajczyk

Journal:
BMC Public Health

Additional file 2 – Differences in 2014's risk perception between individuals who responded to both surveys and individuals who responded to the first survey only

	First survey completed (N=974) n (%)	Second survey completed		
		No (N=354) n (%)	Yes (N=620) n (%)	p-value
**Affective response**				
**Percentage of participants who are worried...**				
...about EVD.	281 (28.9)	112 (31.6)	169 (27.3)	0.17
...that in the next three months people might arrive in Germany who are identified as infected persons after their entry.	736 (76.0)	261 (74.4)	475 (77.0)	0.40
...that individual persons might be infected with the Ebola virus in Germany during the next six months.	579 (59.9)	223 (63.7)	356 (57.7)	0.077
...that in the next six months Ebola could spread in the general population of Germany similar to how it is spreading currently in West Africa.	33 (3.4)	13 (3.7)	20 (3.2)	0.83
**Knowledge score**, median (interquartile range)	7.0 (6.0, 9.0)	7.0 (5.0, 9.0)	7.0 (6.0, 9.0)	0.0085
**Cognitive response: likelihood of infection**				
**Percentage of participants who think that they have a personal risk of acquiring Ebola…**				
…at work.	72 (8.6)	24 (7.5)	48 (9.3)	0.46
... in public transport.	156 (16.9)	60 (17.9)	96 (16.4)	0.61
... in public places (school, childcare …) or public events.	171 (17.9)	70 (20.2)	101 (16.6)	0.20
…at an airport in Germany.	347 (37.4)	125 (37.4)	222 (37.4)	1.00
…as a patient in a German hospital.	147 (15.3)	56 (16.1)	91 (14.8)	0.66
…at a doctor's office in Germany.	158 (16.4)	59 (16.9)	99 (16.2)	0.84
…during a travel to affected countries.	493 (73.1)	190 (74.5)	303 (72.3)	0.59
**Cognitive response: personal impact**				
**Percentage of participants who would…**				
...avoid public events and crowded places.	149 (15.4)	56 (16.0)	93 (15.1)	0.77
...avoid using public transport.	138 (14.3)	47 (13.4)	91 (14.7)	0.64
...avoid physical contact with other people.	312 (32.3)	103 (29.4)	209 (33.9)	0.17
...pay more attention to hygiene (e.g. wash hands more often).	630 (65.1)	219 (62.6)	411 (66.6)	0.23
...wear a face mask outside of my home.	12 (1.2)	5 (1.4)	7 (1.1)	0.93
...not want to be admitted to the same hospital.	463 (47.9)	159 (45.4)	304 (49.4)	0.27
...not visit friends admitted to the same hospital.	254 (26.3)	89 (25.4)	165 (26.8)	0.70

	First survey completed (N=974) n (%)	Second survey completed No (N=354) n (%)	Second survey completed Yes (N=620) n (%)	p-value
**Cognitive response: coping efficacy**				
**Percentage of participants who support specific measure to prevent the spread of EVD to Europe**				
Provide information on EVD for all travelers coming from affected areas and advice in case of developing symptoms	933 (97.0)	339 (96.6)	594 (97.2)	0.72
Get personal information of all travelers coming from affected areas and control their health three weeks long	662 (68.8)	241 (68.7)	421 (68.9)	1.00
Forbid return transport of Germans getting infected during assistance intervention in West Africa	90 (9.3)	38 (10.8)	52 (8.5)	0.28
Forbid bringing EVD patients for treatment to Germany	242 (25.3)	91 (26.1)	151 (24.8)	0.72
Measure temperature of all travelers coming from affected countries when they arrive in Europe with subsequent quarantine for those with high temperature	569 (59.7)	217 (62.0)	352 (58.4)	0.30
Measure temperature of all travelers coming from affected countries when they leave Africa with subsequent quarantine for those with high temperature	519 (54.5)	197 (56.4)	322 (53.3)	0.38
Mandatory quarantine for all volunteers returning from assistance intervention in West Africa	359 (37.6)	126 (36.0)	233 (38.5)	0.48
Visa ban for people from affected countries	164 (17.0)	60 (17.0)	104 (16.9)	1.00
Forbid traveling from Germany to affected countries in Africa	153 (15.9)	58 (16.5)	95 (15.5)	0.73
Compulsory vaccination against Ebola for all inhabitants of affected countries as soon as a vaccine is available	828 (85.9)	297 (84.6)	531 (86.6)	0.44
